# Supplementary material for: Molecular programming modulates hepatic lipid metabolism and adult metabolic risk in the offspring of obese mothers in a sex-specific manner
Source: Commun Biol. 2022 Oct 4;5:1057. doi: 10.1038/s42003-022-04022-3 (PMC9532402; doi:10.1038/s42003-022-04022-3)
Supplement: Supplementary file 2 — Supplementary Information [file 42003_2022_4022_MOESM2_ESM.pdf]

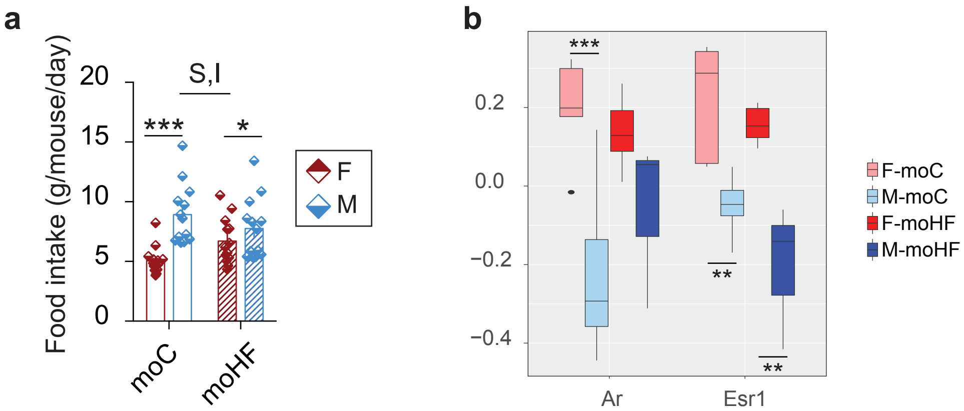

Figure S1. Offspring food intake and relative expression level of sex hormone genes in offspring´s liver.
a Average food intake in offspring (g/mouse/day), F-moC (red open bars), M-moC (blue open bars), F-moHF (red stripped bars) and M-moHF (blue stripped bars); **b** Box plots showing expression (RPKM, log10) of *Esr1* and *Ar* genes. Data are presented as mean ± sem. Differences between sexes (F *versus* M) were determined by two-tailed unpaired t-test corrected for multiple comparisons using the Holm–Sidak method, with alpha = 5.000%. *, M *versus* F, $p < 0.05$; ** $p < 0.01$.
For **b** F-moC (n =5), M-moC (n = 5), F-moHF (n =6) and M-moHF (n = 3).

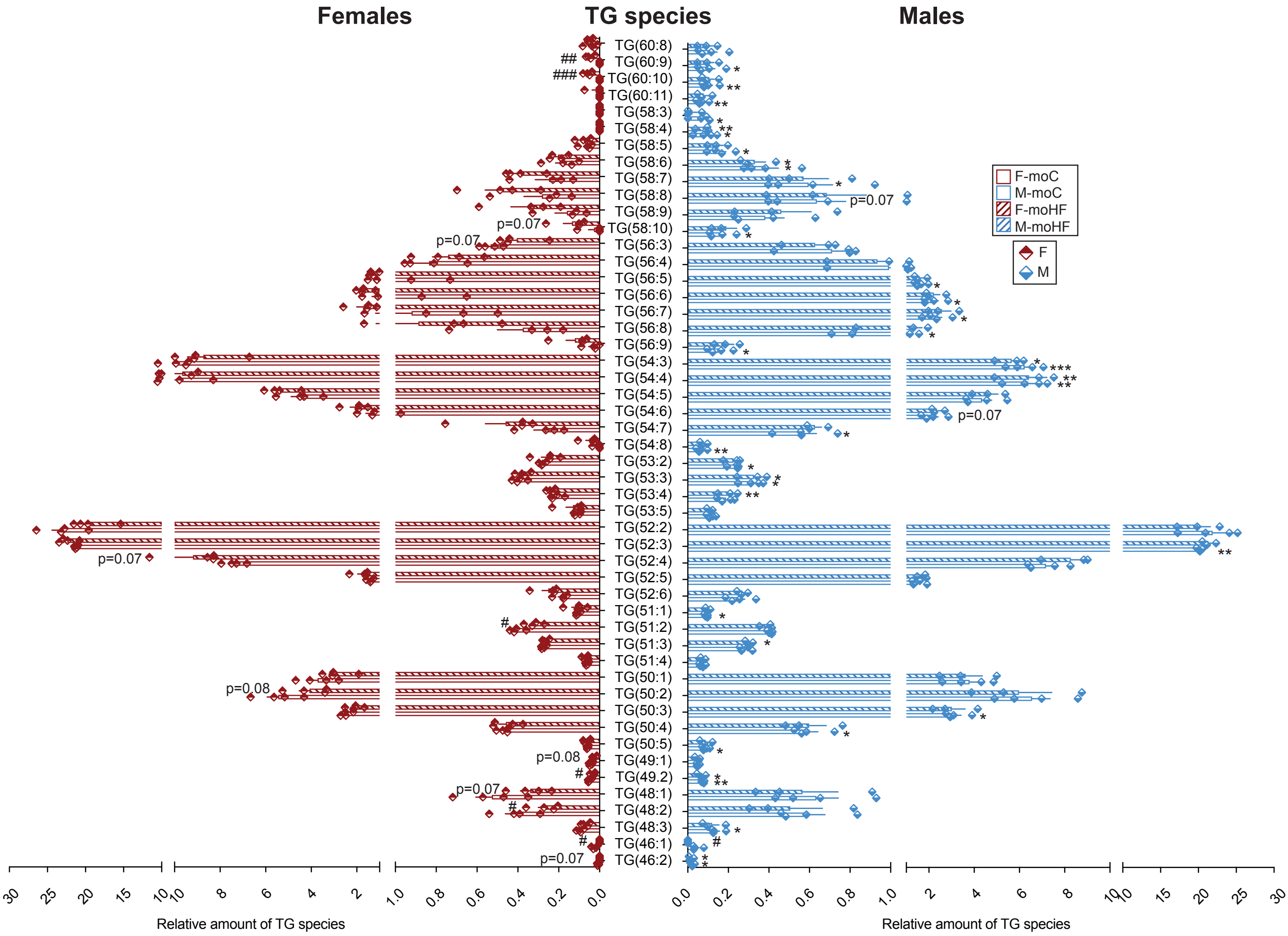

Figure S2. Relative abundance of TG species in offspring's liver detected by LC-MS. Data are presented as mean ± sem. Differences between two groups (sexes, F *versus* M; maternal diet moC *versus* moHF) were determined by two-tailed unpaired t-test corrected for multiple comparisons using the Holm–Sidak method, with alpha = 5.000%. F-moC (n =4), M-moC (n = 4), F-moHF (n =4) and M-moHF (n = 3). *, M *versus* F and #, moHF *versus* moC, $p < 0.05$; ** or ##, $p < 0.01$; *** or ###, $p < 0.001$.

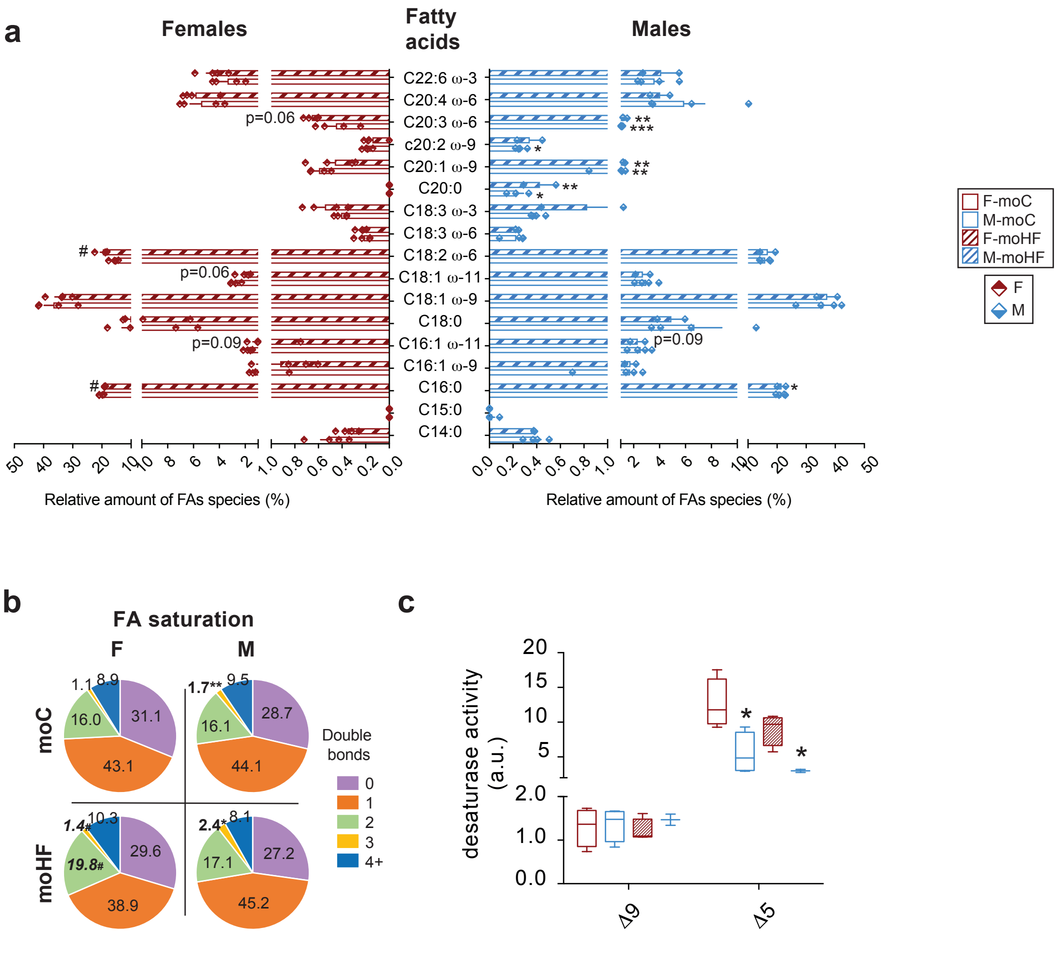

Figure S3. Relative abundance of fatty acid (FA) species in offspring.
a Relative abundance of fatty acid (FA) species contained into the total TG and PL; **b** Pie charts of the FA saturation profile in F-moC, M-moC, F-moHF and M-moHF; **c** Delta 9 and delta 5 desaturase activity in F-moC (red open box), M-moC (blue open box), F-moHF (red stripped box) and M-moHF (blue stripped box). For **a-c** F-moC (n =4), M-moC (n = 4), F-moHF (n =4) and M-moHF (n = 3). Data are presented as mean ± sem. Differences between two groups (sexes, F *versus* M; maternal diet, moC *versus* moHF) were determined by two-tailed unpaired t-test corrected for multiple comparisons using the Holm–Sidak method, with alpha = 5.000%.
*, M *versus* F and #, moHF *versus* moC, $p < 0.05$; **, $p < 0.01$; ***, $p < 0.001$.

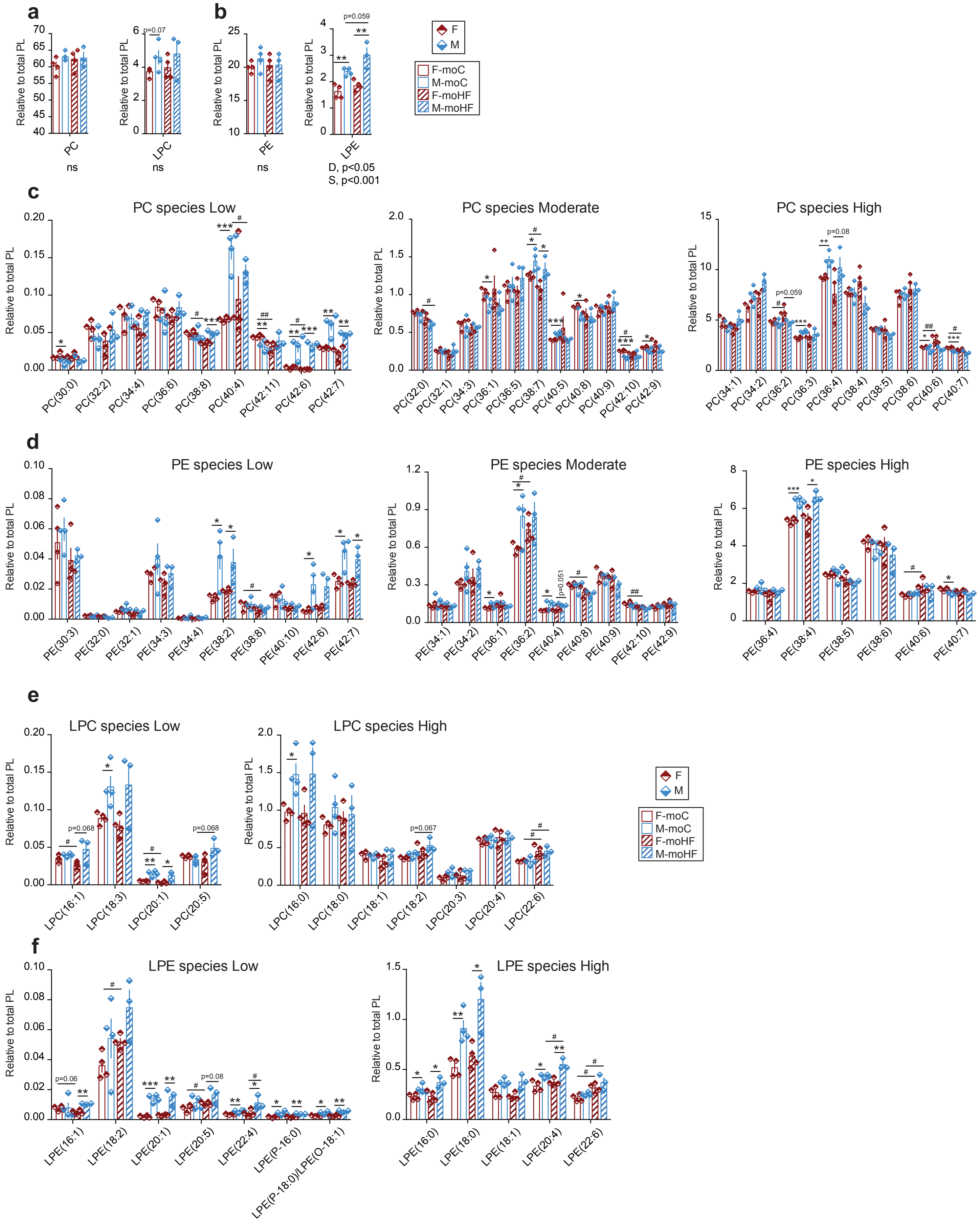

Figure S4. Relative abundance of PL lipid classes and species in offspring liver. Relative levels of **a** PC and LPC; **b** PE and LPE lipid classes; Low, moderate and high relative levels of **c** PC and **d** PE species; Low and high relative levels of **e** LPC and **f** LPE species. For **a-f** F-moC (n =4), M-moC (n = 4), F-moHF (n =4) and M-moHF (n = 3). Data are presented as mean ± sem. Two-way ANOVA (sex (S), mother diet (D), interaction (I) between sex and diet, and (ns) for not significant) followed by Tukey's multiple comparisons test when significant ($p < 0.05$). Differences between two groups (sexes, F *versus* M; maternal diet, moC *versus* moHF) were determined by two-tailed unpaired t-test corrected for multiple comparisons using the Holm–Sidak method, with alpha = 5.000%. *, M *versus* F and #, moHF *versus* moC, $p < 0.05$; **or ##, $p < 0.01$; ***, $p < 0.001$.

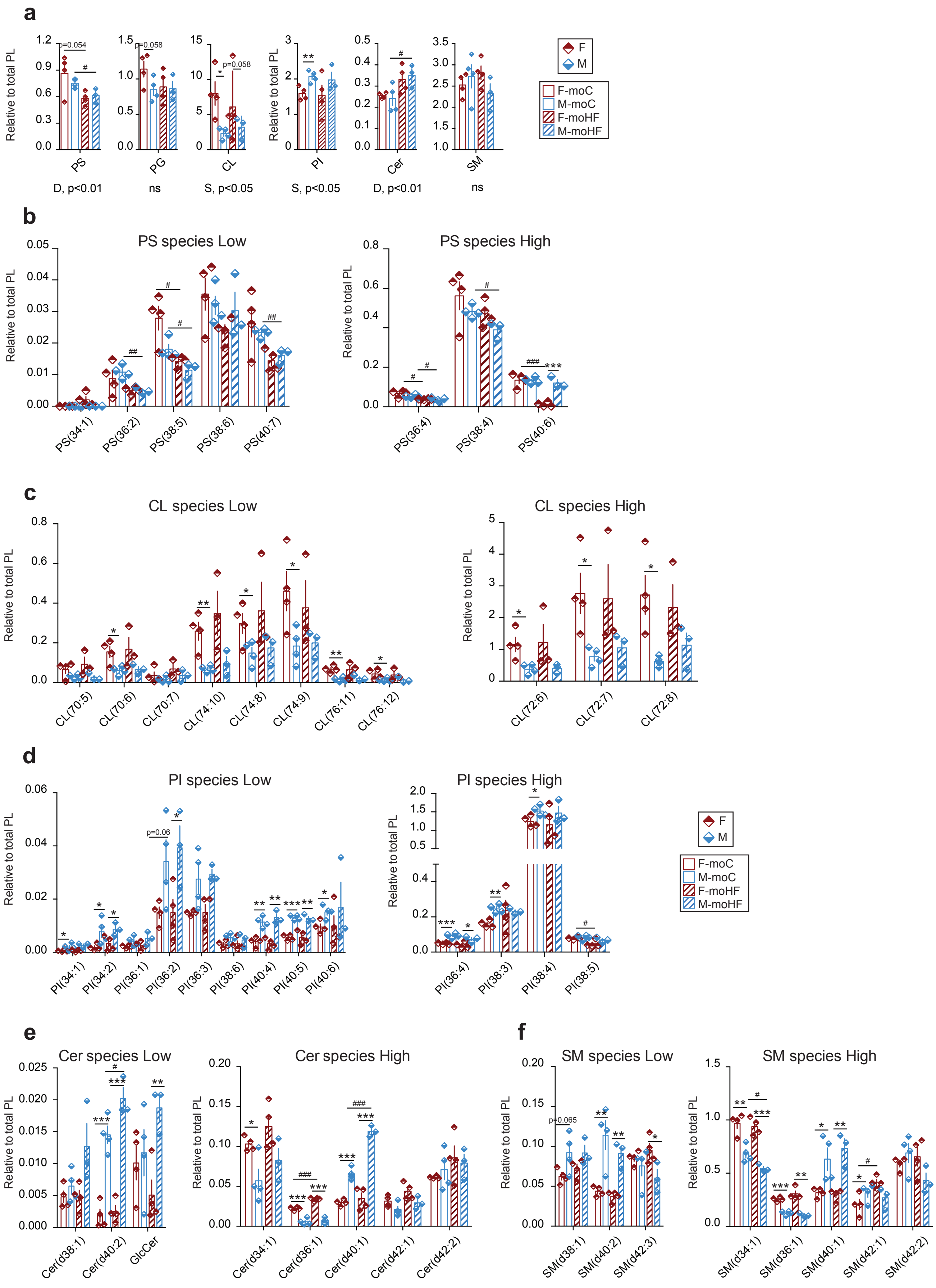

Figure S5. Relative abundance of PL lipid classes and species in liver. Relative levels of **a** PS, PG, CL, PI, Cer and SM lipid classes; Low and high relative levels of **b** PS; **c** CL; **d** PI; **e** Cer; and **f** SM species. Data are presented as mean ± sem. Two-way ANOVA (sex (S), mother diet (D), interaction (I) between sex and diet, and (ns) for not significant) followed by Tukey's multiple comparisons test when significant ($p < 0.05$). Differences between two groups (sexes, F versus M; maternal diet, moC versus moHF) were determined by two-tailed unpaired t-test corrected for multiple comparisons using the Holm–Sidak method, with alpha = 5.000%. *, M versus F and #, moHF versus moC, $p < 0.05$; ** or ##, $p < 0.01$; *** or ###, $p < 0.001$.

Table S1: Differentially expressed (DE) genes of the glucose and insulin pathways in offspring’s liver.

Pathway	SEX		DIET	
	moC	moHF	Females	Males
Glycolysis / Gluconeogenesis	Acss2; Adh4; Ldha; Pklr; Aldh7a1; Dlat; Aldh3b3; Adh1; Akr1a1; Aldh1b1; Adh5; G6pc; Pck1	Adh4; Pdhb; Aldob; Aldh1b1	Acss2; Pklr; Gapdh; Dlat; Bpgm	Aldh1b1
MAPK signaling pathway	Ntrk2; Egfr; Fgfr3; Mknk2; Tnfrsf1a; Rac2; Jund; Ddit3; Hspb1; Hspa8	Rasgrp2; Ntrk2; Egfr; Nras; Mapk3; Tnfrsf1a; Il1r1; Hspb1	Nf1; Ntrk2; Mknk2	-
cAMP signaling pathway	Gabbr2; Rapgef4; Calm2; Rac2; Pik3r1; Creb3l3; Sox9; Acox1; Pde4b; Atp2a2	Rapgef4; Gabbr2; Calm2; Mapk3; Acox1; Pde4b	Calm2; Pde3b	Fxyd1
PI3K-Akt signaling pathway	Efna1; Egfr; Fgfr3; Prlr; Fn1; Vtn; Gng11; Sgk1; Hsp90aa1; Hsp90ab1; Pck1; G6pc; Ccnd1; Cdkn1a; Ccnd3; Creb3l3; Rxra; Pik3r1	Egfr; Nras; Mapk3; Ghr; Prlr; Fn1; F2r; Prkaa2; Eif4e; Them4; Hsp90aa1; Gsk3b; Gys2; Ccnd1; Ywhaz	Efna1; Col4a1; Vtn; Ccnd1; Rxra	Hsp90ab1
AMPK signaling pathway	Hmgcr; Fasn; Acaca; Ccnd1; Scd1; Lepr; Creb3l3; Pck1; G6pc; Srebf1; Stradb; Adipor2; Pik3r1; Acacb	Ccnd1; Gys2; Stradb; Prkaa2; Pparg	Acaca; Hmgcr; Scd1; Ccnd1; Adipoq; Fasn; Lep; Acacb	-
Insulin signaling pathways & Type II diabetes mellitus	Pik3r1; Ppp1r3b; Calm2; Pygl; Srebf1; Acaca; Acacb; Fasn; Pklr; G6pc; Pck1; Mknk2; Slc2a2; Creb3l3; Rapgef4; Tnfrsf1a; Slc27a2; Pygl; Mlxipl	Gsk3b; Gys2; Calm2; Prkaa2; Eif4e; Nras; Mapk3; Rapgef4; Tnfrsf1a	Ppp1r3c; Calm2; Pde3b; Acaca; Acacb; Fasn; Pklr; Mknk2; Slc27a5; Adipoq	-

DE genes analysis from the KEGG pathways using FDR<0.1 and p-value< 0.05 as a cut-off in offspring’s liver. SEX: DE genes between female and male offspring born from control diet fed mothers (moC) and HFD fed mothers (moHF). DIET: DE genes in response to MO in female and male offspring. For F-moC, n=5; M-moC, n=5; F-moHF, n=6 and M-moHF, n=3.

Table S2: Differentially expressed (DE) genes of the lipid metabolism pathways in offspring's liver.

	SEX		DIET	
Pathway	moC	moHF	Females	Males
Biosynthesis of unsaturated fatty acids	Acot3; Elovl6; Scd1; Acot4; Acaa1b; Acox1; Acaa1a; Fads1; Acot1; Baat; Pecr	Acot3; Acaa1b; Hsd17b12; Acox1	Elovl6; Scd1; Acot3; Fads1; Fads2	-
Fatty acid biosynthesis	Fasn; Acaca; Acacb; Acsl5; Acsl3; Acsl1	Acsl4	Acaca; Acacb; Fasn; Acsl3; Acsl4; Acsl5	-
Fatty acid degradation	Cyp4a12a; Adh4; Cyp4a12b; Acat2; Aldh7a1; Acsl5; Acaa1b; Acsl3; Echs1; Acox1; Acsl1; Cyp4a32; Cpt2; Acaa1a; Adh1; Acadm; Aldh1b1; Acadsb; Adh5	Cyp4a12a; Cyp4a12b; Adh4; Acaa1b; Cyp4a32; Aldh1b1; Acox1; Acsl4	Acat2; Acsl3; Acsl4; Acsl5	Aldh1b1
Fatty acid elongation	Elovl3; Acot3; Elovl6; Acot4; Echs1; Acot1	Elovl3; Acot3; Hsd17b12	Elovl6; Acot3	-
Fat digestion and absorption	Acat2; Plpp3; Scarb1; Abcg8; Plpp1; Dgat2; Abcg5	Apob	Acat2; Scarb1	-
Non-alcoholic fatty liver disease (NAFLD)	Tnfrsf1a; Pik3r1; Rxra; Srebf1; Mlxipl; Pklr; Lepr; Adipor2; Xbp1; Ddit3; Casp7; Ndufa4; Cox4i1; Cox6a1	Tnfrsf1a; Gsk3b; Prkaa2; Xbp1; Eif2s1; Cycs; mt-Co1	Rxra; Pklr; Lep; Adipoq; Cyp2e1; Uqcr11; Cox8b;	-
PPAR signaling pathway	Fabp5; Cyp4a12a; Scp2; Pltp; Cyp4a12b; Scd1; Me1; Ubc; Acsl5; Acaa1b; Acsl3; Acox1; Acsl1; Cyp4a32; Cpt2; Acaa1a; Pck1; Plin5; Cyp8b1; Slc27a2; Acadm; Rxra; Angptl4	Cyp4a12a; Cyp4a12b; Scp2; Acaa1b; Fabp5; Cyp4a32; Acox1; Me1; Ucp1; Pparg; Acsl4	Me1; Fabp5; Scd1; Adipoq; Acsl5; Acsl3; Lpl; Pltp; Slc27a5; Rxra; Cyp7a1; Acsl4; Fabp4; Fads2	-
Adipocytokine signaling pathway	Tnfrsf1a; Acsl1; Acsl3; Acsl5; Lepr; Pck1; G6pc; Rxra; Adipor2; Acacb	Tnfrsf1a; Acsl4; Prkaa2	Acsl4; Acsl3; Acsl5; Lep; Rxra; Adipoq; Acacb	-
Regulation of lipolysis in adipocytes	Pik3r1; Plaat3		Fabp4; Pde3b	-

DE genes analysis from the KEGG pathways using FDR<0.1 and p-value< 0.05 as a cut-off in offspring's liver. SEX: DE genes between female and male offspring born from control diet fed mothers (moC) and HFD fed mothers (moHF). DIET: DE genes in response to MO in female and male offspring. For F-moC, n=5; M-moC, n=5; F-moHF, n=6 and M-moHF, n=3.

Table S3: Differentially expressed (DE) genes of the inflammatory pathways in offspring's liver.

Pathway	SEX		DIET	
	moC	moHF	Females	Males
Apoptosis	Tnfrsf1a; Cflar; Casp6; Casp7; Dffa; Ddit3; Ctsd; Ctsh; Ctsl; Ctss; Pik3r1	Tnfrsf1a; Casp6; Cycs; Eif2s1; Ctsb; Ctsc; Nras; Mapk3	Ctsb; Ctsc; Ctsd	-
Natural killer cell mediated cytotoxicity	H2-T23; H2-K1; Rac2; Pik3r1	Mapk3; Tyrobp; Fcer1g; Zap70; Nras	-	-
T cell receptor signaling pathway	Pik3r1	Zap70; Mapk3; Nck1; Nras; Gsk3b	-	-
B cell receptor signaling pathway	Pik3r1; Rac2	Mapk3; Gsk3b; Nras	-	-
TNF signaling pathway	Tnfrsf1a; Cflar; Creb3l3; Casp7; Il15; Pik3r1	Tnfrsf1a; Mapk3; Cxcl1; Mmp14	Ifi47	-
Leukocyte trans endothelial migration	Pik3r1; Rapgef4; Rac2	Rapgef4	Cldn1	-
Chemokine signaling pathway	Ccl9; Pik3r1; Rac2; Gng11; Elmo1	Cxcl1; Cxcl9; Ccl9; Nras; Mapk3; Gsk3b; Rasgrp2	Stat1	-
NF-kappa B signaling pathway	Tnfrsf1a; Cflar	Zap70; Il1r1; Tnfrsf1a; Lbp	Lbp	-

DE genes analysis from the KEGG pathways using FDR<0.1 and p-value< 0.05 as a cut-off in offspring's liver. SEX: DE genes between female and male offspring born from control diet fed mothers (moC) and HFD fed mothers (moHF). DIET: DE genes in response to MO in female and male offspring. For F-moC, n=5; M-moC, n=5; F-moHF, n=6 and M-moHF, n=3.

Table S4: Differentially expressed (DE) genes of the hepatocellular carcinoma in offspring's liver.

Pathway	SEX		DIET	
	moC	moHF	Females	Males
Cell cycle	Ccnd1; Ccnd3; Hdac1; Cdkn2c; Cdkn1c; Cdkn1a	Ccnd1; Gsk3b; Ywhaz; Anapc11; Stag2	Ccnd1	-
Chemical Carcinogenesis	Cyp2c40; Gstp1; Cyp2c68; Cyp2c37; Cyp3a41a; Ephx1; Cyp3a44; Sult1a1; Cyp2c23; Cyp3a16; Cyp2c38; Gsta2; Cyp2c54; Gsta4; Cyp3a41b; Cyp2c39; Cyp2c70; Gstt2; Gsta1; Gstp2; Mgst1; Gstt3; Gstk1; Sult2a1; Sult2a3; Hsd11b1; Ugt2b5; Ugt1a2; Ugt1a6a; Ugt1a9; Ugt1a10; Ugt1a7c; Ugt1a5; Ugt2b1; Ugt1a6b; Ugt1a1; Ugt2b36; Cyp2b13; Cyp2b9; Aldh3b3; Adh1; Adh4; Adh5; Kyat3; Kyat1	Cyp2c40; Gstp1; Cyp2c38; Sult1a1; Cyp3a44; Cyp3a41a; Ephx1; Cyp2c68; Cyp2c70; Cyp3a16; Cyp2c37; Cyp2c39; Gstt1; Mgst3; Mgst1; Gstm7; Gstt3; Sult2a1; Sult2a3; Hsd11b1; Ugt2b5; Ugt1a2; Ugt1a6a; Ugt1a9; Ugt1a10; Ugt1a7c; Ugt1a5; Ugt2b1; Ugt1a6b; Ugt1a1; Ugt2b34; Ugt2b36; Ugt2b38; Ugt2b35; Cyp2b13; Cyp2b9; Adh4	Cyp2c23; Cyp2c50; Cyp2c37; Cyp2c54; Cyp1a2; Cyp2c38; Cyp2c29; Gsta4; Ugt1a2; Ugt1a9; Ugt1a10; Ugt1a7c; Ugt1a6b; Ugt1a1; Cyp2e1; Cyp2b9	Cyp2c37; Cyp2c50; Cyp2c54; Ugt2b5; Ugt2b36
Choline metabolism in cancer	Egfr; Pik3r1; Rac2; Chka; Chpt1; Plpp1; Plpp3	Egfr; Mapk3; Nras; Chpt1		
MicroRNAs in cancer	Ccnd1; Egfr; Abcb1a; Cdkn1a; Fgfr3	Egfr; Ccnd1; Pdcd4; Nras	Ccnd1; Slc45a3	-
Mineral absorption	Slc11a2; Trf; Slc39a4; Steap2; Mt1	Trf; Slc39a4	Trf	-
mTOR signaling pathway	Pik3r1	Eif4e; Mapk3; Prkaa2		-
Notch signaling pathway	Psenen; Hdac1	Psen2; Hes1		
Oocyte meiosis	Ar; Calm2	Cpeb2; Mapk3; Ywhaz; Anapc11; Calm2	Calm2	-
p53 signaling pathway	Ccnd1; Cdkn1a; Ccnd3	Ccnd1; Cycs	Ccnd1	
Pathways in cancer	Ccnd1; Wnt5b; Agtr1a; Gng11; Fn1; Pik3r1; Cdkn1a; Egfr; Fgfr3; Rac2; Rxra; Hdac1; Elob; Epas1; Ar; Hsp90aa1; Hsp90ab1	Ccnd1; Gsk3b; F2r; Agtr1a; Fn1; Rasgrp2; Egfr; Nras; Mapk3; Dapk1; Pparg; Spi1; Cycs; Fh1; Hsp90aa1	Ccnd1; Col4a1; Stat1; Rxra	Hsp90ab1
Proteoglycans in cancer	Ccnd1; Esr1; Pik3r1; Egfr; Cdkn1a; Vtn; Fn1; Gpc1; Wnt5b; Ctsl	Ccnd1; Mapk3; Esr1; Nras; Ddx5; Egfr; Cav1; Cav2; Pdcd4; Sdc1; Fn1	Ccnd1; Dcn; Cav1; Cav2; Sdc1; Vtn	

Retinol metabolism	Cyp2b13; Cyp2c40; Cyp2a4; Cyp4a12a; Cyp2c68; Cyp2a5; Rdh11; Cyp2c37; Aox3; Ugt2b5; Cyp3a41a; Rdh16; Adh4; Cyp4a12b; Cyp3a44; Ugt2b1; Cyp2c23; Cyp3a16; Aox1; Cyp2c38; Ugt1a5; Cyp2b9; Cyp2c54; Cyp3a41b; Cyp4a32; Ugt1a2; Ugt1a10; Cyp2c39; Ugt2b36; Ugt1a6b; Hsd17b6; Lrat; Ugt1a6a; Adh1; Ugt1a7c; Ugt1a9; Dhrs4; Cyp2c70; Ugt1a1; Aldh1a1; Retsat; Dhrs9; Adh5	Cyp2b9; Cyp2b13; Cyp2a4; Cyp2c40; Cyp4a12a; Aox3; Cyp2c38; Ugt2b5; Cyp3a44; Cyp4a12b; Adh4; Cyp3a41a; Cyp2c68; Ugt2b38; Cyp2c70; Cyp3a16; Ugt2b36; Aldh1a1; Ugt2b1; Aox1; Cyp2c37; Ugt2b35; Cyp2a5; Rdh9; Aldh1a7; Ugt1a5; Cyp2c39; Ugt1a1; Ugt1a2; Ugt1a10; Ugt1a9; Ugt1a6b; Ugt1a7c; Dhrs4; Ugt2b34; Dhrs3; Ugt1a6a; Cyp4a32	Cyp2a4; Rdh16; Cyp2c23; Cyp2c50; Cyp2c37; Rdh9; Cyp2c54; Cyp1a2; Ugt1a1; Hsd17b6; Rdh11; Dhrs3; Cyp2b9; Aldh1a7; Cyp2c38; Cyp2a12; Ugt1a6b; Cyp2c29; Ugt1a7c; Ugt1a2; Ugt1a10; Ugt1a9	Cyp2c37; Cyp2c50; Ugt2b5; Cyp2c54; Ugt2b36

DEG analysis using FDR<0.1 and p-value< 0.05 as a cut-off, that belong to the selected KEGG pathways in offspring's liver. SEX: DEG between sexes in offspring born from CD fed mothers (moC) and HFD fed mothers (moHF). DIET: DEG in response to MO in female and male offspring. For F-moC, n=5; M-moC, n=5; F-moHF, n=6 and M-moHF, n=3.
