# Supplementary material for: Molecular programming modulates hepatic lipid metabolism and adult metabolic risk in the offspring of obese mothers in a sex-specific manner
Source: Commun Biol. 2022 Oct 4;5:1057. doi: 10.1038/s42003-022-04022-3 (PMC9532402; doi:10.1038/s42003-022-04022-3)
Supplement: Supplementary file 3 — Description of Additional Supplementary Files [file 42003_2022_4022_MOESM3_ESM.pdf]

Description of Additional Supplementary Files

File name: Supplementary Data 1

Description: The source data behind the RNAseq graphs in the paper.
