# Supplementary material for: Molecular programming modulates hepatic lipid metabolism and adult metabolic risk in the offspring of obese mothers in a sex-specific manner
Source: Commun Biol. 2022 Oct 4;5:1057. doi: 10.1038/s42003-022-04022-3 (PMC9532402; doi:10.1038/s42003-022-04022-3)
Supplement: Supplementary file 5 — Reporting Summary [file 42003_2022_4022_MOESM5_ESM.pdf]

nature portfolio

Corresponding author(s): Marion Korach-André

Last updated by author(s): Aug 30, 2022

Reporting Summary

Nature Portfolio wishes to improve the reproducibility of the work that we publish. This form provides structure for consistency and transparency in reporting. For further information on Nature Portfolio policies, see our Editorial Policies and the Editorial Policy Checklist.

Statistics

For all statistical analyses, confirm that the following items are present in the figure legend, table legend, main text, or Methods section.

n/a	Confirmed	
☐	☒	The exact sample size (*n*) for each experimental group/condition, given as a discrete number and unit of measurement
☐	☒	A statement on whether measurements were taken from distinct samples or whether the same sample was measured repeatedly
☐	☒	The statistical test(s) used AND whether they are one- or two-sided<br>*Only common tests should be described solely by name; describe more complex techniques in the Methods section.*
☒	☐	A description of all covariates tested
☐	☒	A description of any assumptions or corrections, such as tests of normality and adjustment for multiple comparisons
☐	☒	A full description of the statistical parameters including central tendency (e.g. means) or other basic estimates (e.g. regression coefficient) AND variation (e.g. standard deviation) or associated estimates of uncertainty (e.g. confidence intervals)
☒	☐	For null hypothesis testing, the test statistic (e.g. *F*, *t*, *r*) with confidence intervals, effect sizes, degrees of freedom and *P* value noted<br>*Give P values as exact values whenever suitable.*
☒	☐	For Bayesian analysis, information on the choice of priors and Markov chain Monte Carlo settings
☒	☐	For hierarchical and complex designs, identification of the appropriate level for tests and full reporting of outcomes
☒	☐	Estimates of effect sizes (e.g. Cohen's *d*, Pearson's *r*), indicating how they were calculated

Our web collection on statistics for biologists contains articles on many of the points above.

Software and code

Policy information about availability of computer code

Data collection	Magnetic resonance imaging and spectroscopy: Paravision<br>Lipidomic analysis: for fatty acids (gas chromatography – mass spectrometry (GC-MS)), for TG and PL (high-performance liquid chromatography (HPLC) – mass spectrometry (LC-MS) system)
Data analysis	Magnetic resonance imaging: fiji http://fiji.sc<br>Magnetic resonance spectroscopy: LCModel http://s-provencher.com/pub/LCModel/manual/manual.pdf<br>Lipidomics: excel and GraphPad Prism softwares<br>Statistical analysis and graphs: Graphpad prism v7.1.2<br>Heatmaps, bubble charts and PCA plot: R 4.0.2<br>RNA-seq analysis: DEseq2, R 4.0.2

For manuscripts utilizing custom algorithms or software that are central to the research but not yet described in published literature, software must be made available to editors and reviewers. We strongly encourage code deposition in a community repository (e.g. GitHub). See the Nature Portfolio guidelines for submitting code & software for further information.

Data

Policy information about availability of data

All manuscripts must include a data availability statement. This statement should provide the following information, where applicable:

- Accession codes, unique identifiers, or web links for publicly available datasets
- A description of any restrictions on data availability
- For clinical datasets or third party data, please ensure that the statement adheres to our policy

The raw data generated for lipidomics, and RNA sequencing are available as described below
For VAT and SAT RNA sequencing, SRA data: PRJNA662930
For Liver RNA sequencing, SRA data: PRJNA723771
For the lipidomic, SRA data: https://figshare.com/s/ac91b57eaa0f5c560d3d

Human research participants

Policy information about studies involving human research participants and Sex and Gender in Research.

Reporting on sex and gender	NA
Population characteristics	NA
Recruitment	NA
Ethics oversight	NA

Note that full information on the approval of the study protocol must also be provided in the manuscript.

Field-specific reporting

Please select the one below that is the best fit for your research. If you are not sure, read the appropriate sections before making your selection.

☒ Life sciences ☐ Behavioural & social sciences ☐ Ecological, evolutionary & environmental sciences

For a reference copy of the document with all sections, see nature.com/documents/nr-reporting-summary-flat.pdf

Life sciences study design

All studies must disclose on these points even when the disclosure is negative.

Sample size	The sample size for each experimental group are provided in the method section and in the figure legends.
Data exclusions	No data was excluded.
Replication	Each animal was evaluated in a follow-up experiment for in vivo analysis which allowed us to identify potential problem in each individual. All ex vivo data available in the present study were from biological replicates and any attempt at replication was successful. For RNA-seq we used triplicates as technical replicates.
Randomization	Each offspring born from either obese or lean mothers was randomized for diet after weaning. We used offspring from six different mothers to randomized our data.
Blinding	During the execution of both in vivo and ex vivo experiments, we opted for randomized experimental design (random selection of the animals for in vivo and ex vivo experiments) and labeled the animals/tubes with numbers to blind group allocation.

Reporting for specific materials, systems and methods

We require information from authors about some types of materials, experimental systems and methods used in many studies. Here, indicate whether each material, system or method listed is relevant to your study. If you are not sure if a list item applies to your research, read the appropriate section before selecting a response.

Materials & experimental systems

n/a	Involved in the study	
☒	☐	Antibodies
☒	☐	Eukaryotic cell lines
☒	☐	Palaeontology and archaeology
☐	☒	Animals and other organisms
☒	☐	Clinical data
☒	☐	Dual use research of concern

Methods

n/a	Involved in the study	
☒	☐	ChIP-seq
☒	☐	Flow cytometry
☒	☐	MRI-based neuroimaging

Animals and other research organisms

Policy information about studies involving animals; ARRIVE guidelines recommended for reporting animal research, and Sex and Gender in Research

Laboratory animals	Mice with C57bl/6J background
Wild animals	The current study did not involve wild animals
Reporting on sex	The current study considered both sexes and focused on the study of sex-differences
Field-collected samples	The current study did not involve samples collected from the field.
Ethics oversight	All animal procedures were approved by the local Ethical Committee of the Swedish National Board of Animal Experiments

Note that full information on the approval of the study protocol must also be provided in the manuscript.
